# Designing Comprehensive Public Health Surveillance for Enteric Fever in Endemic Countries: Importance of Including Different Healthcare Facilities

**DOI:** 10.1093/infdis/jiy191

**Published:** 2018-07-27

**Authors:** Senjuti Saha, Maksuda Islam, Shampa Saha, Mohammad Jamal Uddin, Hafizur Rahman, Rajib Chandra Das, Md Hasan, Md Ruhul Amin, Mohammed Hanif, Mohammad Shahidullah, Manzoor Hussain, Samir K Saha

**Affiliations:** 1 Child Health Research Foundation, Department of Microbiology, Dhaka Shishu Hospital, Bangladesh; 2 Bangladesh Institute of Child Health, Dhaka Shishu (Children) Hospital, Bangladesh; 3 Bangabandhu Sheikh Mujib Medical University, Bangladesh; 4 Popular Diagnostic Center, Bangladesh

**Keywords:** enteric fever, paratyphoid, phoid, surveillance

## Abstract

**Background:**

Designing comprehensive surveillance to generate credible burden estimates of enteric fever in an endemic country can be challenging because care-seeking behavior is complex and surveillance in different healthcare facilities may lead to documentation of different epidemiological characteristics.

**Methods:**

We conducted retrospective surveillance in 3 healthcare facilities to identify culture-confirmed enteric fever cases in Dhaka, Bangladesh, from January 2012 through December 2016. The study settings included (1) hospital in-patient department (IPD), (2) hospital out-patient department (OPD), and (3) private consultation center OPD. We analyzed the cases to understand their distribution, age ranges, and antibiotic susceptibility patterns across the settings.

**Results:**

Of the 1837 culture-confirmed enteric fever cases, 59% (1079 of 1837) were OPD cases. Children with enteric fever hospitalized in the IPDs were younger than children seeking care at the hospital OPD (median age: 45 vs 60 months) or private OPD (median age: 45 vs 72 months). Multidrug resistance rates were slightly higher in hospital IPD cases than in private OPD cases (26% vs 24%).

**Conclusions:**

In each facility, we identified different epidemiological characteristics, and lack of consideration of any of these may result in misinterpretation of disease burden, identification of different age groups, and/or antibiotic susceptibility patterns.

Enteric fever continues to be a major life-threatening disease and was estimated to cause 17 million illnesses and 178000 deaths in 2015 [1, 2]. Etiologies of enteric fever are *Salmonella enterica* serovar Typhi (typhoid) and *Salmonella enterica* serovar Paratyphi A, B, and C (paratyphoid). The majority of burden occurs in low- and middle-income countries, particularly in Asia and sub-Saharan Africa [3–7]. Enteric fever was one of the major killers in the pre-antibiotic era, but use of effective antibiotics have reduced mortality rates from 30% to <1% [8]. In recent years, however, emergence of widespread antimicrobial resistance is threatening effective treatment and if the right intervention strategies are not put in place soon, increases in mortality rates in the near future is inevitable. A major hurdle in implementation of evidence-based treatment and prevention policies is that disease characteristics and estimates have been mostly derived from sporadic, incidence studies, usually using a single healthcare facility.

Designing an inclusive surveillance system for systematic collection of data and filling knowledge gaps on enteric fever can be difficult in resource-poor countries, where most of the burden exists, because of the complexity in care-seeking behavior and treatment strategies [9]. In Bangladesh, for example, with no national healthcare system in place, patients pay out of their pockets and decide whether and where to seek care, primarily based on the cost of treatment and ability of the respective patient/caregiver. Besides patients hospitalized with enteric fever in in-patient departments (IPD) of hospitals, a large number of patients seek care and are treated at the out-patient departments (OPD), often with no records left at the hospitals. Therefore, IPD-based surveillance may not provide a good estimate of disease burden and may not be representative of the epidemiological characteristics of the OPD cases, and hence data collected from both IPDs and OPDs should be analyzed. Furthermore, a sizeable number of patients in the community seek care in the soaring number of community-based private consultation centers, and these cases are often missed by hospital-based surveillance systems. Data collected from hospitals may differ from that collected from private consultation centers. For instance, previous data from Bangladesh have revealed a higher rate of multidrug resistance of *Salmonella* Typhi strains identified in a hospital compared with those identified in a private consultation center [10]. Consequently, to generate an overall picture and credible burden estimates of a disease in a resource-poor endemic country, it is important to recognize the complexity in care-seeking behavior when designing a surveillance system.

We retrospectively analyzed blood culture-confirmed enteric fever cases captured in 3 different facilities defined according to care-seeking behavior of the population in Dhaka, Bangladesh, from January 2012 through December 2016. Our study included (1) hospital IPD-based surveillance to capture hospitalized enteric fever cases, (2) hospital OPD-based surveillance to capture cases who were not advised and/or did not seek hospitalization and received out-patient-based treatment, and (3) private consultation center OPD-based surveillance. By comparing epidemiological characteristics, primarily age distribution and antibiotic susceptibility patterns, of cases captured through these 3 settings, we elucidate the importance of including each component in a comprehensive surveillance study for enteric fever.

## MATERIAL AND METHODS

### Study Sites and Enteric Fever Case Enrollment

This retrospective study included 3 surveillance sites: (1) Dhaka Shishu (Children) Hospital (DSH), (2) Shishu Shasthya (Child Health) Foundation Hospital (SSFH), and (3) Popular Diagnostic Center ([PDC] headquarter branch, Dhanmondi). Dhaka Shishu (Children) Hospital is the largest pediatric hospital in the country with 640 beds and provides primary to tertiary care to children from all over the country. This government-aided private hospital treats 37% of patients free of cost. Shishu Shasthya (Child Health) Foundation Hospital provides primary care and is the second largest pediatric hospital in Bangladesh with 200 beds for children, 5% of which are dedicated to those unable to pay for care. Popular Diagnostic Center is one of the largest private consultation centers in Bangladesh and caters to a higher socioeconomic class than the hospitals and to patients of all age groups.

For (1) hospital IPD-based surveillance to capture hospitalized enteric fever cases, we obtained data from IPDs of DSH and SSFH, (2) hospital OPD-based surveillance, we obtained data from OPD of DSH (OPD of SSFH was not included because data were not available) and (3) private consultation center OPD-based surveillance, we collected data from OPD of PDC. Treatment of OPD cases, at DSH and PDC, is home-based, where the attending physician prescribes oral antibiotic and sends the patients home.

All blood cultures performed on children aged 2 months–16 years at each setting between January 2012 and December 2016 were analyzed to extract cases that yielded growth of *Salmonella* Typhi or *Salmonella* Paratyphi. A case was defined as a patient with positive blood culture for either of the 2 organisms. Date of blood culture and age of patient were abstracted from hospital or clinic records.

### Etiology Detection and Antibiogram

Data on all *Salmonella* Typhi and Paratyphi isolates were obtained from DSH and SSFH laboratory records. In DSH and SSFH, routine blood cultures were performed using standard methods described earlier [11]. All *Salmonella* Typhi and Paratyphi isolates from blood culture in PDC were reconfirmed at the DSH laboratory. Antibiotic susceptibility tests were conducted for ampicillin, cotrimoxazole, and chloramphenicol using disc diffusion methods and interpreted according to the 2015 Clinical and Laboratory Standards Institute guidelines.

### Data Analysis

All data were entered into EpiData (The EpiData Association, Odense, Denmark) and analyzed using Stata 13 (STATACorp, College Station, TX). A descriptive analysis aimed to understand isolation rates, age distribution, and antibiotic susceptibility patterns of *Salmonella* Typhi and Paratyphi A in the different settings was performed. Categorical associations were evaluated using Pearson’s χ^2^ test and Fisher’s exact test.

### Ethical Clearance

The protocols for this retrospective study were approved by the ethics review committees of the Bangladesh Institute of Child Health, DSH, Bangladesh.

## RESULTS

### Blood Culture-Confirmed Enteric Fever Cases Identified Between January 2012 and December 2016

#### Hospital In-Patient Department-Based Surveillance

A total of 7834 blood cultures were performed from children aged 2 months–16 years hospitalized in the IPDs of DSH (n = 4506) and SSFH (n = 3328), 12% (958 of 7834) of which were positive for significant bacterial growth (n = 630 in DSH, n = 328 in SSFH) (Table 1). Of these, 79% (758 of 958) were positive for *Salmonella* Typhi/Paratyphi A (73%, 457 of 630, in DSH; 92%, 301 of 328, in SSFH), consisting of 656 (87%) cases of Typhi and 102 (13%) cases of Paratyphi A.

**Table 1.** Summary Of Characteristics of Culture-Confirmed Enteric Fever Cases (2 Months–16 Years) Identified in Each Healthcare Setting Between January 2012 and December 2016

Characteristics	Hospital IPD		Hospital OPD	Private OPD
	DSH	SSFH	DSH	PDC
Total no. of blood cultures performed	4506	3328	4592	NA
No. of growth positive blood cultures	630 (14%)	328 (10%)	615 (13%)	NA
No. of *Salmonella* Typhi/Paratyphi positive cases	457 (73%)	301(92%)	583 (95%)	496
Median age (months)	46	42	60	72
MDR rate of *Salmonella* Typhi	24%	28%	23%	24%

Abbreviations: DSH, Dhaka Shishu (Children) Hospital; IPD, in-patient department; MDR, multidrug resistant; NA, not available; OPD, out-patient department; PDC, Popular Diagnostic Center; SSFH, Shishu Shasthya (Child Health) Foundation Hospital.

#### Hospital Out-Patient Department-Based Surveillance

Among a total of 4592 blood cultures from children aged 2 months–16 years visiting the OPD of DSH, 13% (615 of 4592) were culture positive for significant bacterial growth (Table 1). Of these, 95% (583 of 615) were positive for *Salmonella* Typhi/Paratyphi A, including 477 (82%) *Salmonella* Typhi cases and 106 (18%) *Salmonella* Paratyphi A cases.

#### Private Consultation Center Out-Patient Department-Based Surveillance

The total number of blood cultures performed in PDC was not available, and therefore the culture positivity rates for significant bacterial growth and for *Salmonella* Typhi/Paratyphi A could not be calculated. In the 5-year study period, 496 blood cultures yielded *Salmonella* Typhi (n = 396) Paratyphi A (n = 100) from children aged 2 months–16 years.

**Figure 1.**
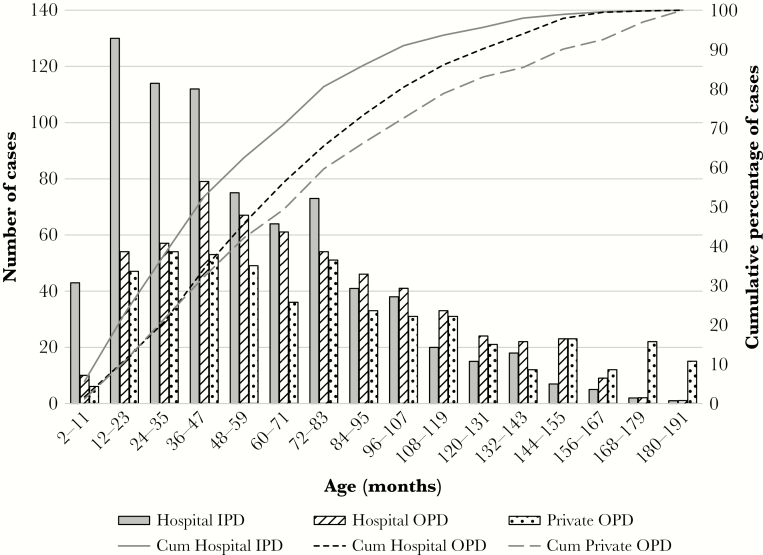
Age distribution of culture-confirmed enteric fever cases (2 months–16 years) identified in each healthcare setting between January 2012 and December 2016. Abbreviations: IPD, in-patient department; OPD, out-patient department; Cum, cumulative.

Taken together, a total of 1837 blood culture-confirmed cases of enteric fever were identified in the 3 healthcare settings (Figure 1). Culture positivity rate for enteric fever cases is significantly higher in hospital OPD than in hospital IPDs (95% vs 79%; *P* < .001). Among the 1040 culture-confirmed cases identified in DSH, where we were able to obtain data from both OPD and IPD, 56% were identified in the OPD (n = 583) of DSH.

### Age Distribution of Culture-Confirmed Enteric Fever Cases Across Healthcare Settings

#### Hospital In-Patient Department-Based Surveillance

For IPD cases (n = 758), median age was 45 months (interquartile range [IQR], 72–25). The median age among DSH IPD cases (n = 457) was slightly older at 46 months, compared with 42 months among SSFH IPD cases (n = 301). Overall, 63% of cases were under 5 years (Figure 1).

#### Hospital Out-Patient Department-Based Surveillance

In the enteric fever cases identified among children visiting DSH OPD, median age was 60 months (IQR, 96–36) and 46% of cases were under 5 years. If enteric fever cases from only DSH are considered, from where both OPD and IPD data were available, children admitted in the IPD are also significantly younger than children seeking care at the OPD. Median age of children with enteric fever in the DSH IPD was 46 months, and 62% of children was less than 5 years, whereas median age of children in the DSH OPD was 60 months, and 46% children were less than 5 years (median age: 46 vs 60 months; *P* < .001) (Figure 1).

#### Private Consultation Center Out-Patient Department-Based Surveillance

The median age of PDC cases aged 2 months to 16 years was 72 months (IQR, 108–36), and 42% of cases were under 5 years (Figure 1). Median age of children with enteric fever visiting the private consultation center OPD was significantly higher than those seeking care at the IPDs of DSH and SSFH (72 vs 45 months; *P* < .001) or the OPD of DSH (72 vs 60 months; *P* = .010).

### Antimicrobial Susceptibility Patterns of *Salmonella* Typhi/Paratyphi A Isolates Across Healthcare Settings

#### Hospital In-Patient Department-Based Surveillance

Information on antibiotic susceptibility patterns was available for 99% (750 of 758) of IPD cases, including 451 cases identified in the IPD of DSH and 299 in the IPD of SSFH (Table 1). For *Salmonella* Typhi, multidrug-resistant ([MDR] resistance to chloramphenicol, ampicillin, and cotrimoxazole) rate was 26% (167 of 651); 24% (96 of 399) for DSH and 28% (71 of 252) for SSFH. By contrast, no (0 of 99) Paratyphi A isolate was MDR.

#### Hospital Out-Patient Department-Based Surveillance

Antimicrobial resistance patterns were available for 99% (582 of 583) of OPD cases (Table 1). Among *Salmonella* Typhi isolates, 23% (111 of 477) were MDR; no (0 of 105) MDR *Salmonella* Paratyphi A isolate was identified.

#### Private Consultation Center Out-Patient Department-Based Surveillance

Antibiotic susceptibility patterns were available for 95% (376 of 396) of Typhi isolates and 97% (97 of 100) of Paratyphi A isolates (Table 1). Among *Salmonella* Typhi isolates, 24% (90 of 376) were MDR; no (0 of 97) MDR *Salmonella* Paratyphi A isolate was found. Proportion of MDR isolates at SSFH and DSH IPD (26%, 167 of 651) compared with PDC (24%, 90 of 376) was higher, but the difference was not significant (*P* = .5).

## DISCUSSION

In South Asia, enteric fever is endemic and *Salmonella* Typhi/Paratyphi A make up for three fourths of all isolates obtained from blood cultures from all patients >2 months of age [12–14]. Enteric fever can be prevented by improving water, sanitation, and hygiene and with use of effective vaccines, but to institute such guidelines or for introduction of vaccines, credible data on disease burden and other epidemiological characteristics are critical. To date, the most common approach to collect data has been through studying cases admitted as in-patients to hospitals or through active community-based surveillance systems. Both of these approaches have certain limitations [9]. In the case of hospital IPD-based surveillance, for example, it is easy to underestimate disease burden because a lot of sick people seek care at the OPD of public hospitals and private OPD-based health centers, where they are prescribed treatment and sent home with minimum and/or no hospital records. On the other hand, community-based active surveillance often captures cases of small geographical areas with little diversity in epidemiological variables, such as socioeconomic status and demographics. Furthermore, in resource-poor countries, with no national health system and tracking methods in place, there is no one center where populations of certain geographical area(s) seek care; it is entirely up to patient’s or caregiver’s discretion where the patient will seek care. Therefore, we need to be careful and thorough when designing surveillance studies in endemic countries such as Bangladesh. In this study, we retrospectively analyzed 1837 blood culture-confirmed enteric fever cases that were captured from hospital IPD and OPD and private consultation center OPD. We compared (1) blood culture positivity rates for *Salmonella* Typhi/Paratyphi, (2) age distribution, and (3) antibiotic susceptibility patterns of culture-confirmed enteric fever cases obtained across the 3 different healthcare facilities. Our aim is to guide informed design of comprehensive enteric fever surveillance systems in endemic, low-resource settings.

In DSH, the largest pediatric hospital of Bangladesh, of the 1040 culture-confirmed enteric fever cases, 56% were identified in the OPD. Studies conducted in Indonesia and Nepal have also reported high rates of culture-positive enteric fever in OPD patients compared with IPD patients within the same hospital [15–17]. This suggests that surveillance that does not include the OPD of hospitals underestimates the burden of enteric fever, because most cases are not hospitalized but treated in the OPD and sent home.

We also found significant differences in age distribution of patients seeking care at the different settings. Younger children are more likely to be hospitalized with enteric fever, and older children are more likely to be treated in the OPD. Overall, enteric fever cases identified in the pediatric hospitals was younger than those in the private consultation center. An IPD-based surveillance study, specifically in pediatric hospitals, will underestimate the disease burden in older children, which may have implications in designing vaccination strategies (such as vaccine catch-up programs) and/or calculating expected vaccine impact. On the other hand, a recent study conducted in a general hospital in Nepal that serves patients of all ages depicted the median age of patients with typhoid to be 16 years, which underestimates the burden in young children [18].

Finally, antibiotic susceptibility patterns were also different across healthcare settings, where hospital IPDs are likely to capture higher rates of MDR, misrepresenting the overall scenario. Multidrug-resistant rate amongst *Salmonella* isolates was 24% in PDC, and it was 28% in the IPD of SSFH. Although the difference we found was not statistically significant, a previous study from Bangladesh reported a greater difference: 42% MDR *Salmonella* Typhi isolates in IPD of DSH vs 17% in OPD of PDC in 1994 [10]. A higher rate of MDR is still noticed in IPD in this study, and overall rates are lower. With shifting trends of antimicrobial resistance, different facilities may capture different snapshots of resistance at different times, and if all facilities are not considered, misinterpretations are inevitable.

An important limitation of the study that should be considered is that the study was not designed to actively identify suspect enteric fever cases; blood culture results were retrospectively abstracted from hospital and clinic records. Obtaining blood cultures was dependent on the discretion of the treating physician, and this is likely to differ between facilities; not all patients with fever may have been advised blood culture by the treating physicians. Despite this limitation, we were able to identify a large number of culture-confirmed enteric fever cases and demonstrated that enteric fever cases identified in each of the 3 setting has different characteristics. Another limitation is that none of the surveillance sites used here has a denominator because they were not population-based, which prohibits incidence calculation. This can be overcome if the data can be linked to a denominator using the low-cost hybrid approach proposed by Luby et al [9]. This approach combines the existing laboratory diagnosis data conducted in healthcare centers with data on healthcare utilization patterns in the catchment population to generate incidence estimates.

## CONCLUSIONS

With enteric fever becoming an increasingly important disease, recently, with investments such as the Coalition against Typhoid supported by the Bill and Melinda Gates Foundation, there has been renewed interest among the global community. On January 3, 2018, the World Health Organization prequalified the first typhoid conjugate vaccine [19]. When evidence-based decisions for introduction of recently prequalified typhoid conjugate vaccine are imminent, it is very important to consider how the evidence is generated and to carefully plan vaccine impact studies. The Sabin Vaccine Institute, with support from the Bill and Melinda Gates Foundation, established a hospital-based enteric fever surveillance network in Asia—the Surveillance for Enteric Fever in Asia Project (SEAP)—to enable systematic collection of data and fill knowledge gaps on impact of severe enteric fever. This multicountry, multisite surveillance study in Bangladesh, Nepal, and Pakistan is the first of its kind. In 2016, it began a prospective surveillance study with defined inclusion criteria for enteric fever and includes all 3 healthcare settings discussed here: hospital IPDs and OPDs and private consultation center OPDs. The SEAP has also integrated community-based surveillance of healthcare facility utilization into its surveillance platform to generate incidence estimates. This will allow comprehensive data collection on enteric fever, generate precise knowledge on care-seeking behavior in specified catchment areas, and overcome the limitations of the study presented here. If successful, SEAP will reduce knowledge gaps, provide credible disease burden estimates, and generate baseline data for vaccine impact studies. The model used by SEAP may provide a useful example and valuable lesson on how a well designed study can go a long way to improve global health.

## Notes

Presented in part: American Society of Tropical Medicine and Hygiene Conference, November 2017, Baltimore, MD.

## Notes

***Acknowledgments.*** We thank Mr. Hafizur Rahman for technical assistance with etiology detection and antibiogram. We are grateful to Popular Diagnostic Center for sharing *Salmonella* Typhi/Paratyphi A isolates and data with us.

***Finacial support.*** This work was partially supported by the Invasive Bacterial Vaccine Preventable Diseases Surveillance Study coordinated by the World Health Organization and the Child Health Research Foundation, Bangladesh.

***Supplement sponsorship.*** This article is part of the supplement “Surveillance for Enteric Fever in Asia Project,” sponsored by the Sabin Vaccine Institute.

***Potential conflicts of interest.*** S. K. S. has received grants from GlaxoSmithKline, Sanofi Pasteur, and Pfizer. All authors have submitted the ICMJE Form for Disclosure of Potential Conflicts of Interest. Conflicts that the editors consider relevant to the content of the manuscript have been disclosed.
